# Cancer stem cell markers in adenocarcinoma of the salivary glands - reliable prognostic markers?

**DOI:** 10.1007/s00405-020-06389-7

**Published:** 2020-10-03

**Authors:** Jennifer L. Spiegel, Mark Jakob, Marie Kruizenga, Saskia Freytag, Mattis Bertlich, Martin Canis, Friedrich Ihler, Frank Haubner, Julia Kitz, Bernhard G. Weiss

**Affiliations:** 1 Department for Otorhinolaryngology, LMU Klinikum, Marchioninistr, Ludwig-Maximilians-Universität München, 81377 Munich, Germany; 2 Department of Otorhinolaryngology, Georg August University, University Hospital Göttingen, Robert-Koch-Str. 40, 37075 Göttingen, Germany; 3 Molecular Medicine, Harry Perkins Institute of Medical Research, Perth, WA 6009 Australia; 4 German Centre for Vertigo and Balance 508 Disorders, LMU Klinikum, Marchioninistr. 15, Ludwig-Maximilians-Universität , 81377 Munich, Germany; 5 Institute of Pathology, University Hospital Göttingen, Georg August University, Robert-Koch-Str. 40,, 37075 Göttingen, Germany

**Keywords:** Salivary glands, Adenocarcinoma, Prognostic marker, Cancer stem cell markers, CSC, Salivary gland malignoma

## Abstract

**Purpose:**

Adenocarcinoma of the salivary glands is of low incidence and a broad range of histopathological subtypes. Cancer stem cell markers (CSC) might serve as novel prognostic parameters. To date, only a few studies examined the expression of CSC in adenocarcinoma of the salivary glands with diverging results. To further investigate the reliability in terms of prognostic value, a histopathological analysis of CSCs on a cohort of patients with adenocarcinomas of the major salivary glands was performed.

**Methods:**

Tumor samples of 40 consecutive patients with adenocarcinoma of the major salivary gland treated with curative intend at one tertiary center were stained with the CSCs ALDH1, BMI-1, CD44, Nanog, and SOX2. Expression of these markers was correlated with clinicopathological parameters and survival estimates.

**Results:**

Correlation of high expression of ALDH1 with higher grading (*p* < 0.001) and high expression of CD44 with the localization of the neoplasm (*p* = 0.05), larger tumor size (*p* = 0.006), positive pN-category (*p* = 0.023), and advanced UICC stage (*p* = 0.002) was found. Furthermore, high expression of SOX2 correlated with a negative perineural invasion (*p* = 0.02). No significant correlation of any investigated marker with survival estimates was observed.

**Conclusion:**

In conclusion, our study did not find a significant correlation of the investigated CSCs with survival estimates in adenocarcinoma of the major salivary glands. Recapitulating the results of our study in conjunction with data in the literature, the CSCs ALDH1, BMI-1, CD44, Nanog, and SOX2 do not seem to serve as reliable prognostic parameters in the treatment of adenocarcinoma of the salivary glands.

**Electronic supplementary material:**

The online version of this article (10.1007/s00405-020-06389-7) contains supplementary material, which is available to authorized users.

## Introduction

Malignoma of the salivary glands exhibits a low incidence of 2.5–3.0 cases per 100,000 people per year [1]. Together with a broad range of more than 20 histological subtypes, and different localization, the investigation of this disease seems challenging [2]. However, a multimodal treatment-regimen with radical resection of the tumour, neck dissection, and adjuvant radiotherapy is recommended, and has shown superior survival in comparison to resection alone [3]. Clinical parameters like histopathological entity, grading, tumour size, and perineural invasion have proven helpful to estimate prognosis in malignoma of the salivary glands [4]. In cases of recurrence or inoperability, treatment options are limited and mostly chemotherapy in terms of a palliative care setting remain [5]. With a disease so rare combined with a possibly poor prognosis, further investigations about the pathogenesis of those tumour entities is required to improve treatment concepts.

Cancer stem cells are known to exhibit infinite properties of self-renewal, reconstitution of tumour-heterogeneity and maintenance of tumour growth [6]. Since the discovery of cancer stem cells, the involvement of certain markers as key regulators in oncologic diseases has been investigated thoroughly throughout the past decades [7]. In the head and neck region, cancer stem cell markers (CSC) are suspected to play a role in oncogenesis, as well as progression and prognosis of the disease [8]. ALDH1 serves as a marker for both tissue-resident stem cells, as well as cancer stem cells of different tissue types [9], like lung, colon, prostate, pancreatic, endometroid cancer, and head and neck malignoma [10]. BMI-1 is an epigenetic key regulator and influences p53 and Rb proteins, and was found to function as an enhancer for self-renewal in hematopoietic stem cells [11], head and neck tumours, and breast adenocarcinomas [12]. CD44 is known as a pivotal marker for cancer stem cells, and an overexpression in cancer cells with a suspected exhibition of highly malignant and therapeutic resistance properties is reported [13]. Also, a correlation of CD44 overexpression in head and neck squamous cell cancer (HNSCC) was observed [14]. The cancer stem cell marker Nanog serves as a marker for pluripotency in both tissue-resident and cancer stem cells and plays a role in maintaining pluripotency [15]. Nanog is reported to correlate with a promotion of metastasis and poor prognosis in HNSCC [16]. SOX2, together with Nanog, is a transcription factor associated with the maintenance of stem cell pluripotency [17]. A correlation with an aggressive feature is reported in colon cancer, breast cancer, and HNSCC [18].

Evaluation of expression of those markers in malignoma of the salivary glands exists only in a few studies so far, which have shown diverging results in correlation with clinicopathological parameters and survival estimates. Thus, the question arises how reliable those CSCs could be in terms of a prognostic parameter. Therefore, we performed a histopathological analysis on a cohort of patients with adenocarcinomas of the major salivary glands to gain further insights into the promising novel prognostic parameter.

## Material and methods

### Patients and compliance with ethical standards

**Fig. 1:**
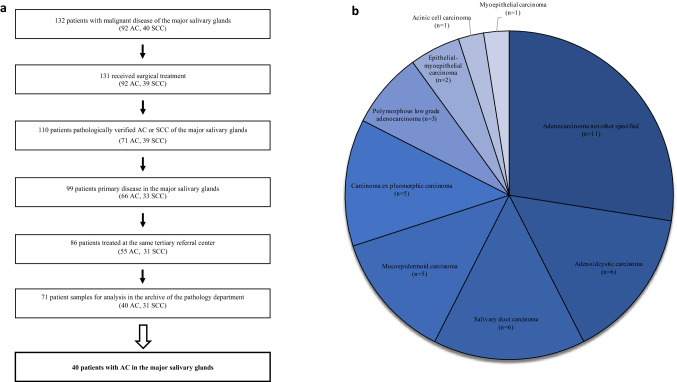
Inclusion criteria and subtypes of adenocarcinomas. **a** 40 patients with adenocarcinoma of the major salivary glands, who received surgical treatment at one tertiary centre were included in the study. **b** 9 different entities of adenocarcinoma were summarized as adenocarcinoma of the major salivary glands, listed in a descending order regarding their share of patients (%). AC, adenocarcinoma; SCC, squamous cell cancer

Retrospective analysis of 132 consecutive patients treated for epithelial malignoma of the salivary glands at one tertiary referral centre, the Department of Otorhinolaryngology, University Medical Centre Göttingen, Georg-August University Göttingen, Germany from 2003 to 2015. Patients treated primarily by surgery in curative intent, and in which an adenocarcinoma of the parotid or submandibular gland was diagnosed pathologically, were included in the analysis (Fig. 1a). 40 patients fulfilled those criteria. The analysis included patients’ and disease characteristics, as well as survival-rates.

The study protocol was performed according to the ethical guidelines of the 2002 Declaration of Helsinki and carried out after approval by the Institutional Review Board and Ethics Committee of the University Medical Centre Göttingen (reference number 2/1/17). All patients gave written consent to the study.

### Immunohistochemistry

After assembly of 40 haematoxylin and eosin-stained slides into a tissue-microarray paraffin block, one-millimetre thick sections were cut and immunohistochemically (IHC) stained. Sections were dewaxed with clearify clearing agent (Agilent, Hamburg, Germany) for 20 min at 65 °C and blocked with EnV FLEX Peroxidase-Blocking Reagent (Agilent, Hamburg, Germany) for 15 min at 97 °C. IHC staining for ALDH-1 (1:200 FLEX + Rabbit, ABCAM, Cambridge, United Kingdom), BMI-1 (1:200 FLEX + Rabbit, CellSignalling, Leiden, Netherlands), Nanog (1:12,800 FLEX + Mouse, CellSignalling, Leiden, Netherlands) and CD44 (1:50 FLEX, CellSignalling, Leiden, Netherlands) was performed with the AutostainerLink 48 (Dako, Hamburg, Germany), incubated for 30 min at room temperature (RT) and incubated another 15 min at RT with the marked polymer EnV FLEX/HRP (Agilent, Hamburg, Germany). The reaction was developed by adding the diaminobenzidine FLEX/DAB + Substrate-Chromogene (Agilent, Hamburg, Germany) and counterstained with haematoxylin (Agilent, Hamburg, Germany) for 3 min at RT. IHC staining with SOX2 (FLEX + Rabbit, Cell Marque, California, USA) was performed with the Dako Omnis (Dako, Hamburg, Germany). Prior to IHC staining with SOX2, the samples were dewaxed with Clearify Clearing Agent for 1 min at 25 °C. Demasking was performed by applying EnV FLEX TRS for 30 min at 97 °C. Then, the antibody for SOX2 was added and incubated for 20 min. For blocking, the samples were incubated with EnV FLEX Peroxidase-Blocking for 3 min. As an enhancer EnV FLEX + Rabbit LINKER was applied. The reaction was developed by adding diaminobenzidine FLEX/DAB + Substrat-Chromogene and then counterstaining with haematoxylin. Between each step, the samples were washed with a buffer solution.

H-score was applied for assessing the extent of immunoreactivity with the following formula:

3 × percentage of strongly staining membrane/cytoplasm/nuclei + 2 × percentage of moderately staining membrane/cytoplasm/nuclei + percentage of weakly staining membrane/cytoplasm/nuclei, giving a range of 0–300 [19]. The H-Score was used by the two examiners in our pathology department. Diverging results were discussed between both examiners.

Calculation of a cutoff-value to define the high or low expression of the markers were performed with a ROC-curve and Youden-Index with the program easyROC version 1.3 [20]. Cutoff-values of the markers were as the following: BMI-1 cutoff at 190; CD44 cutoff at 255; SOX2 cutoff at 30. For calculation of ALDH1 and Nanog lack of staining was considered as a low expression. The clustering heat map was generated via the software Cluster, version 3.0 (Stanford University, Stanford, USA; https://www.encodeproject.org/software/cluster/).

### Statistical analysis

Statistical analysis was performed with the software Statistica, version 13.1 (StatSoft Europe, Hamburg, Germany) with values statistically significant at *p* < 0.05. Statistical differences between groups were calculated by the log-rank test and Mann–Whitney-*U* test. Overall survival (OS), disease-specific-survival (DSS), recurrence-free-survival (RFS) and the local control-rate (LCR) were calculated starting from the date of primary surgery by application of the Kaplan–Meier method. Calculating OS, death for any reason was considered as an event, and patients alive at last follow-up were censored. Regarding DSS, events were defined as death related to the primary tumour alone, and other causes of death were considered as censored. Concerning RFS, local and/or regional recurrences, distant metastasis or death-related to primary diagnosis were considered as events. Whereas, intercurrent-death or death related to secondary primaries, and patients alive without any disease-manifestation accounted for censored observations. In LCR, local recurrences were considered as events. Correlation of expression of CSCs with clinicopathological data was performed by the chi-square test and odds ratio. In the present study three- and 5 year estimates are presented. For multivariate analysis, we used logistic regression to evaluate the effect of clinicopathological variables. To avoid overfitting, we restricted ourselves to fitting clinicopathological variables that had the smallest *p* values in the single variable analysis or were deemed potentially biologically relevant. The model was fitted using the package glm with the software R (Build 3.2.5 for Windows, The R Project for Statistical Computing, https://www.r-project.org/).

## Results

### Patients and disease characteristics

Mean age was 64.4 ± 16.9 years, follow-up was 41.8 ± 42.0 months. Different histopathological subtypes were summarized as adenocarcinomas and are depicted in Fig. 1b. 50% (*n* = 20) were staged a T1-2 tumour, and 52.5% (*n* = 21) did not exhibit a locoregional metastasis at the time of diagnosis. 52.5% (*n* = 21) had a G2-graded adenocarcinoma, and in 90% (*n* = 36) of the patients a R0-resection was reached. Regarding treatment, 50% (*n* = 20) received the resection of the tumour along with a neck dissection and postoperative radiotherapy. A subset of 15 patients (37.5%) received a resection of the tumour with neck dissection, thereof in 10 patients an indication for postoperative radiotherapy was declined by the tumour board, two patients rejected this adjuvant therapy, and in three the radiotherapy was aborted due to complication issues. Three patients received tumour resection alone, of which in one patient no adjuvant therapy was recommended by the interdisciplinary tumour board, and the other two patients rejected the postoperative radiotherapy, as well as a following salvage surgical treatment. Two patients received a resection of the tumour with postoperative radiotherapy. All data regarding patients and disease characteristics are depicted in Table 1.

**Table 1:** Patient and Disease Characteristics with Expression of Cancer Stem Cell Markers

Patient and disease characteristics	*n*	%
Sex		
Female	11	27.5
Male	29	72.5
Primary location		
Parotid gland	28	70
Submandibular gland	12	30
Histological grading		
G1	8	20
G2	21	52.5
G3	11	21.5
pTNM category		
T1–2	20	50
T3–4	20	50
N0	21	52.5
N1	4	10
N2	13	32.5
N3	2	5
UICC stage		
I–II	14	35
III—IV	26	65
Margin status		
R0	36	90
R1	3	7.5
Rx	1	2.5
Pn		
Pn0	26	65
Pn1	14	35
Treatment		
Surgical tumor resection	3	7.5
Surgical tumor resection + ND	15	37.5
Surgical tumor resection + RT	2	5
Surgical tumor resection + ND + RT	20	50
Recurrence	10	25

Expression of cancer stem cell markers	Low expression		High expression	
	*n*	%	*n*	%
ALDH1	36	90	4	10
BMI-1	19	47.5	21	52.5
CD44	28	70	12	30
Nanog	36	90	4	10
SOX2	33	82.5	7	17.5

*CT* chemotherapy, *n*, number of cases, *ND* neck dissection, *Pn* perineural invasion, *R* margin status, *RT* radiotherapy, *TNM* tumor, nodal metastasis, *UICC* International Union Against Cancer (7th edition)

### Immunohistochemistry

**Fig. 2:**
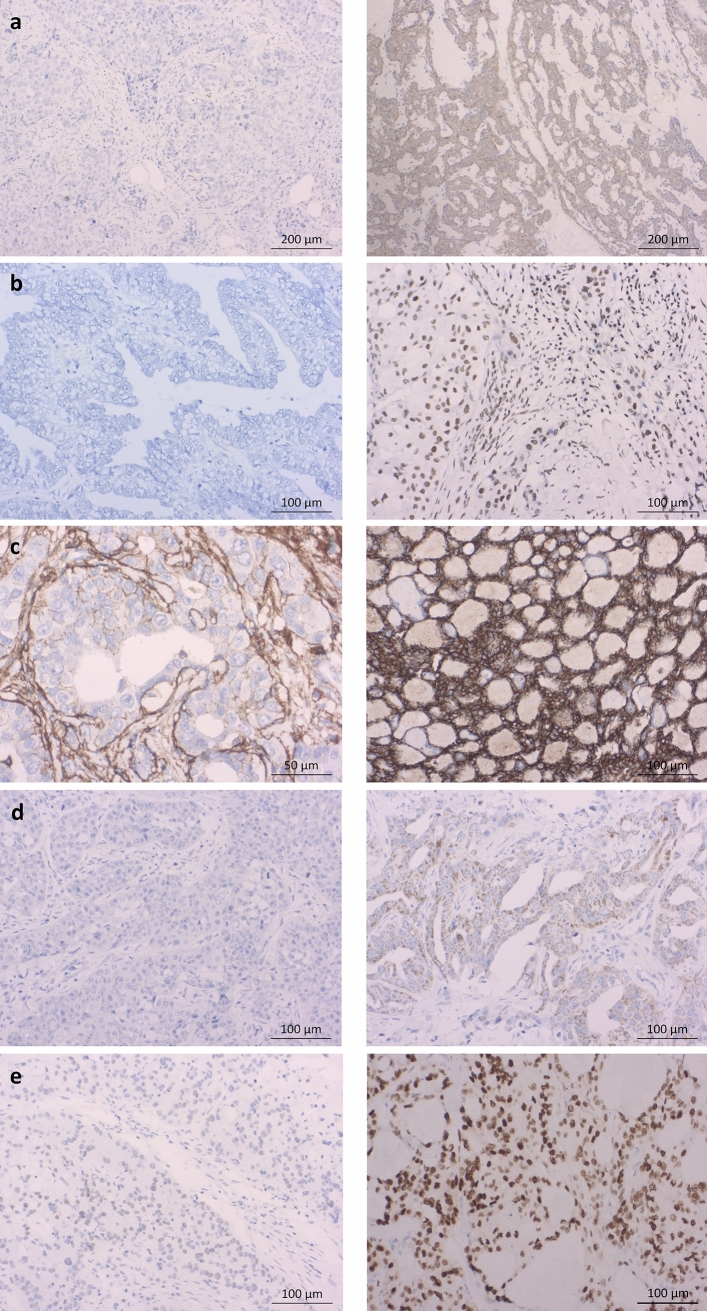
Immunohistochemical staining with low and high expression of the cancer stem cell markers ALDH1, BMI-1, CD44, Nanog, and SOX2. (**a**) Immunostaining with ALDH1: low expression (left; × 10) in a ductal adenocarcinoma, high expression (right; × 10) in an adenoidcystic carcinoma. (**b**) Immunostaining with BMI-1: low expression (left; × 20) in an adenocarcinoma, high expression (right; × 20) in a salivary duct carcinoma. (**c**) Immunostaining with CD44: low expression (left; × 40) in a salivary duct carcinoma, high expression (right; × 20) in an adenocarcinoma. (**d**) Immunostaining with Nanog: low expression (left; × 20) in a mucoepidermoid carcinoma, high expression (right; × 20) in a mucoepidermoid carcinoma. (**e**) Immunostaining with SOX2: low expression (left; × 20) in a mucoepidermoid carcinoma, high expression (right; × 20) in a polymorphous low-grade adenocarcinoma

The complete cohort was analysed for high or low expression of the CSC ALDH1, BMI-1, CD44, Nanog, and SOX2. BMI-1 was highly expressed in 52.5% (*n* = 21) of the cohort, CD44 in 30% (*n* = 12). In terms of expression of ALDH1, Nanog, and SOX2, most of the cohort showed a low expression. Detailed data are shown in Table 1. Exemplary immunohistochemical staining for low and high expression is depicted in Fig. 2a–e.

### Correlation of patient and disease characteristics with CSC

Concerning an association of CSCs with clinicopathological parameters, a strong correlation of ALDH1 expression and higher histopathological grading (G1 vs. G2-3) was observed (*p* < 0.001). Expression of CD44 significantly correlated with the localization of the neoplasm (parotid vs. submandibular gland; *p* = 0.050), a larger tumour size (T1-2 vs. T3-4; *p* = 0.006), positive N-category (*p* = 0.023), and advanced UICC stage III-IV (*p* = 0.002). Regarding a high expression of SOX2, a significant association with a negative perineural invasion was observed (*p* = 0.020). In terms of BMI-1 and Nanog, no correlation was shown within this cohort (Table 2). To find a correlation upon patient and disease characteristics, a hierarchical clustering heat map was performed (Supplemental Fig. 1). By generating the heat map, two groups were identified: Group 1 contained tumour samples with high expression of BMI-1/CD44, and low expression of ALDH1/Nanog/SOX2, which correlated significantly with a higher grading (G1 vs. G2/3; *p* = 0.029). Group 2 was characterised by tumour samples exhibiting low expression of BMI-1/CD44 and high expression of ALDH1/Nanog/SOX2 associated with a lower grading (Supplemental Table 1). Results of the corresponding multivariate analysis are displayed in Supplemental Table 2.

**Table 2:** Correlation of patient and disease characteristics with the expression of cancer stem cell markers

	ALDH1			BMI-1			CD44			Nanog			SOX2		
	Low	High	*p* value	Low	High	*p* value	Low	High	*p* value	Low	High	*p* value	Low	High	*p* value
Sex															
Female	9	2	0.288	5	6	0.873	9	2	0.315	10	1	0.906	8	3	0.479
Male	27	2		14	15		19	10		26	3		24	5	
Age															
< 60 years	14	3	0.166	7	10	0.491	11	6	0.530	16	1	0.455	12	5	0.201
≥ 60 years	22	1		12	11		17	6		20	3		20	3	
Localization															
Parotid gland	25	3	0.818	14	14	0.629	17	11	0.050	24	4	0.168	23	5	0.605
Submandibular gland	11	1		5	7		11	1		12	0		9	3	
Grading															
G1/2	25	4	0.194	12	17	0.208	22	7	0.189	26	3	0.906	21	8	0.051
G3	11	0		7	4		6	5		10	1		11	0	
Grading															
G1	4	4	**< 0.001**	5	3	0.342	7	1	0.227	8	0	0.292	6	2	0.693
G2/3	32	0		14	18		21	11		28	4		26	6	
pT-category															
T1–T2	17	3	0.292	16	4	0.288	18	2	0.006	19	1	0.292	17	3	0.429
T3–T4	19	1		13	7		10	10		17	3		15	5	
pN-category															
Negative	18	3	0.342	12	9	0.199	18	3	0.023	20	1	0.246	18	3	0.342
Positive	18	1		7	12		10	9		16	3		14	5	
Perineural invasion															
Pn0	22	4	0.122	13	13	0.666	18	8	0.885	25	1	0.077	18	8	**0.020**
Pn1	14	0		6	8		10	4		11	3		14	0	
UICC stage															
I–II	11	3	0.077	8	6	0.370	14	0	0.002	13	1	0.658	13	1	0.136
III–IV	25	1		11	15		14	12		23	3		19	7	

*G* grading, *N* nodal status, *Pn* perineural invasion, *T* tumor size, *UICC* International Union Against Cancer (7th edition)

### Correlation of patient and disease characteristics and CSC with survival rates

Three- and 5 year survival-estimates (OS, DSS, RFS, LCR) were correlated with patient and disease characteristics, as well as expression of CSC (Table 3). Concerning histopathological grading, significant differences in both three- and 5 year survival rates between G1-2 and G3 with regard to OS (5 year-estimates: 87.0 vs. 20.2%; *p* < 0.001), DSS (5 year-estimates: 100.0 vs. 38.1%; *p* < 0.001), and RFS (5 year estimates: 83.3 vs. 40.5%; *p* = 0.043) were seen. Comparison of UICC stages showed a significant difference in RFS between UICC I-II and UICC III-IV (5 year-estimates: 90.0 vs. 61.9%; *p* = 0.030). Overall, no statistically significant differences were found regarding survival rates of high and low expression of the examined CSCs. Same findings were observed in the results of hierarchical clustering (Supplemental Table 3).

**Table 3:** Correlation of patient and disease characteristics and expression of cancer stem cell markers with survival Rates

	*n*	OS (%)			DSS (%)			RFS (%)			LCR (%)		
		3 years	5 years	*p* value	3 years	5 years	*p* value	3 years	5 years	*p* value	3 years	5 years	*p* value
Complete cohort	40	71.3	65.8		82.4	82.4		71.9	71.9		84.6	84.6	
Sex													
Female	11	88.9	88.9	0.138	100.0	100.0	0.130	87.5	87.5	0.497	87.5	87.5	0.648
Male	29	64.4	58.0		75.5	75.5		66.1	66.1		83.7	83.7	
Age													
< 60 years	18	76.5	76.5	0.195	8.3	81.3	0.752	75.6	75.6	0.778	86.7	86.7	0.557
≥ 60 years	22	66.2	49.6		83.6	83.6		68.5	68.5		83.1	83.1	
Primary location													
Parotid gland	28	59.7	59.7	0.730	74.5	74.5	0.099	63.8	63.8	0.062	77.3	77.3	0.094
Submandibular gland	12	100.0	85.7		100.0	100.0		90.0	90.0		100.0	100.0	
Histological grading													
G1–2	29	95.7	87.0	**< 0.001**	100.0	100.0	**< 0.001**	83.3	83.3	**0.043**	87.5	87.5	0.421
G3	11	20.2	20.2		38.1	38.1		40.5	40.5		75.0	75.0	
T-category													
T1–T2	20	85.9	73.7	0.298	91.7	9.7	0.142	79.8	79.8	0.139	93.8	93.8	0.166
T3–T4	20	58.1	58.1		73.7	73.7		64.9	64.9		75.7	75.7	
N-category													
N0	21	77.7	77.7	0.330	82.1	82.1	0.978	82.3	82.3	0.146	94.1	94.1	0.075
N +	19	63.1	52.6		82.5	82.5		57.2	57.2		71.9	71.9	
UICC stage													
UICC I–II	14	82.5	82.5	0.355	90.0	90.0	0.326	90.0	90.0	**0.030**	100.0	100.0	0.067
UICC III–IV	26	65	56.9		78.5	78.5		61.9	61.9		75.6	75.6	
Postoperative radiotherapy													
RT +	22	71.7	63.7	0.299	82.5	82.5	0.405	68.3	68.3	0.286	89.1	89.1	0.051
RT −	6	41.7	41.7		50.0	50.0		37.5	37.5		37.5	37.5	
ALDH1													
High	4	100.0	100.0	0.159	100.0	100.0	0.254	100.0	100.0	0.165	100.0	100.0	0.326
Low	36	67.1	61.0		79.4	79.4		67.7	67.7		82.4	82.4	
BMI-1													
High	21	87.4	77.7	0.051	87.4	87.4	0.400	72.7	72.7	0.972	84.0	84.0	0.841
Low	19	54.1	54.1		77.4	77.4		72.0	72.0		84.8	84.8	
CD44													
High	12	75.0	75.0	0.690	81.8	81.8	0.814	63.6	63.6	0.231	71.6	71.6	0.145
Low	28	67.5	57.8		82.1	82.1		75.1	75.1		91.1	91.1	
Nanog													
High	4	66.7		0.936	66.7		0.473	50.0		0.217	75.0		0.498
Low	36	71.6	66.1		84.2	84.2		74.5	74.5		85.5	85.5	
SOX2													
High	7	100.0	75.0	0.184	100.0	100.0	0.192	71.4	71.4	0.613	83.3	83.3	0.854
Low	33	64.1	64.1		77.6	77.6		72.5	72.5		84.8	84.8	

*DSS* disease-specific survival, *G* grading, *LCR* local control rate, *n* number of cases, *N* N-category, *OS* overall survival, *RFS* recurrence-free survival, *RT* radiotherapy, *T* tumor size, *UIC*, International Union Against Cancer, 7th edition;

## Discussion

The current study found no significant differences in survival-rates with regard to the investigated CSCs BMI-1, CD44, ALDH-1, Nanog, and SOX2. Concerning a correlation with disease characteristics, an association of high expression of ALDH1 with grading, high expression of CD44 with localization of the neoplasm, T- and N-category, and UICC stage, as well as high expression of SOX2 with perineural invasion was observed. Regarding IHC, the cohort of adenocarcinoma of the major salivary glands showed a predominantly high expression of BMI-1. Significant lower survival-estimates correlated with a high-grade histopathological disease.

Since salivary gland malignoma is a rather rare entity [1], clinicopathological data is scarce. To date, only 9 studies have examined the prognostic value of CSCs in adenocarcinoma of the major and minor salivary glands (Table 4). Our study is the only one so far to evaluate the correlation of the markers ALDH1, BMI-1, CD44, Nanog, and SOX2 with survival-rates and clinicopathological data in adenocarcinoma of the major salivary glands. The limitations lie in the retrospective nature and small evaluated cohort of the study. To apply a sound statistical comparison, higher numbers would be preferable.

**Table 4:** Reference literature of cancer stem cell markers in adenocarcinoma of the salivary glands

Author	Year	Histological entity	Localisation	*n*	CSC	Findings
Binmadi N., et al*.* [30]	2016	Mucoepidermoid carcinoma	Parotid gland (87%) Submandibular gland (13%)	15	CD44	No significant correlation with prognostic markers
Dai W., et al*.* [39]	2014	Adenoidcystic carcinoma	Not further specified	131	SOX2	*SOX2*: High expression is associated with advanced T category, distant metastasis, OS, and DFS
Destro Rodrigues MFS., et al*.* [22]	2016	Mucoepidermoid carcinoma	Minor/major salivary glands	28	BMI-1 CD44 Oct4 SOX2	Nanog: no correlation with survival rates Oct4/Nanog combined: correlation with perineural invasion
Sedassari BT., et al*.* [40]	2017	Carcinoma ex-pleomorphic adenoma	Parotid gland (50%) Submandibular gland (3%) Minor salivary glands (47%)	30	SOX2	SOX2: high expression is associated with grading, T-category, recurrence rate, distant metastasis, and adverse outcome
Soave D., et al*.* [21]	2013	Adenoidcystic carcinoma (49%) Mucoepidermoid carcinoma (16%) Adenocarcinoma NOS (7%) Basal cell adenocarcinoma (7%) Acinic cell carcinoma (6%) Carcinoma ex-pleomorphic adenoma (6%) Salivary duct carcinoma (4%) Polymorphous low-grade adenocarcinoma (4%)	Minor salivary glands (39%) Parotid gland (38%) Submandibular gland (19%) Sublingual gland (3%)	69	CD24 CD44	CD44: High expression is associated with localization of neoplasm CD24: high expression is associated with clinical stage III/IV, T-category, and N-category CD44/CD24 combined: is associated with the localisation, T-category, and N-category
Wang Y., et al*.* [32]	2011	Adenoidcystic carcinoma	Not further specified	75	CD44v6 Ezrin Ki-67	CD44v6: high expression correlates with distant metastasis, histologic pattern, perineural and vascular invasion, and clinical stage Ezrin: high expression is associated with histologic pattern, tumor diameter, distant metastasis, clinical stage, and poor survival time
Xu W., et al*.* [31]	2017	Mucoepidermoid carcinoma	Minor salivary glands	75	CD44 CD133 SOX2 Nanog	CD44, CD133, Nanog, SOX2 alone: no significant prognostic value CD44/CD133/SOX2 combined: strong prognostic value, correlation with OS
Yi C., et al*.* [26]	2015	Adenoidcystic carcinoma	Not further specified	102	BMI-1 E-cadherin Snail Slug	BMI-1: high expression is associated with worse 5 year MFS
Zhou JH., et al*.* [23]	2013	Adenoidcystic carcinoma	Minor salivary glands and others (42%) Parotid gland (12%) Lacrimal gland (6%) Submandibular gland (5%)	216	ALDH1 Nanog	ALDH1: highly expressed in stromal cells with no correlation in respect to survival estimates

*CSC* cancer stem cell marker, *DFS* disease-free survival, *n* number of patients, *MFS* metastatic-free survival, *OS* overall survival

The cohort of the present study consists of a comparable sample size when looking at other studies which analysed CSCs in adenocarcinomas of the salivary glands [21, 22]. Concerning the distribution of patients and disease characteristics, the data of our study is in line with the literature, where a large cohort of 4068 patients with malignoma of the salivary glands from the National Cancer Database (NCDB) of the American Cancer Society and Commission on Cancer of the American College of Surgeons was investigated with regard to postoperative radiotherapy on survival-estimates [3].

In terms of ALDH1, an association of low expression with higher histopathological grading was observed. Zhou et al. observed a high ALDH1-expression in stromal cells of their cohort of 216 patients with adenoidcystic carcinoma; however, a significant correlation to survival-estimates was not found [23]. Sun et al. showed a high correlation of high ALDH-1-levels in adenoidcystic carcinoma with higher tumorigenic, invasive, and metastatic properties [24]. Regarding other tumour entities, diverging results in terms of a prognostic parameter were found. For example, in endometroid cancer higher ALDH1-expression correlated with longer overall and disease-free survival [25]; whereas, the study group around Chen et al. reported of a positive correlation of ALDH1 expression with a negative outcome in HNSCC [10].

Regarding BMI-1, our cohort exhibited a high expression in half of the cases, without correlation to neither survival-estimates nor clinicopathological parameters. Yi et al. found a high correlation of metastatic disease with expression of BMI-1 in a cohort of 102 patients with adenoid cystic carcinoma [26]. Whereas Destro Rodrigues et al. did not observe any correlation to survival-estimates or prognostic parameters [27]. Concerning other cancer entities, previously Koren et al. found a low expression of BMI-1 in 96 advanced staged non-small cell lung cancer (NSCLC) in comparison to their control group of healthy individuals. Furthermore, they observed a correlation of high BMI-1 with longer progression-free and overall survival in advanced NSCLC patients [28]. Also, an association with high BMI-1-expression was observed in breast cancer, melanoma, gastric cancer, and nasopharyngeal carcinoma [12, 29]. CD44 showed a low expression in the majority of our carcinoma, mucoepidermoid-carcinoma, and other types of adenocarcinoma as investigated in our cohort [21, 27, 30, 31]. Regarding survival-estimates, we did not observe any correlation with CD44 when tested alone. Concerning an association with clinical parameters, a correlation with the tumour localization, tumour size, nodal status, and prognostic UICC stage was observed. Since CD44 is recognized as a pivotal marker for the cancer stem cells [13], a correlation with malignant clinicopathological characteristics and the worse prognosis seems consistent. The group of Xu et al. observed a strong correlation with a significantly increased overall survival in patients showing high expression of CD44, CD133, and SOX2 combined either all together, or CD44 with one of the other both markers. An association of one of the three markers alone with clinicopathological data was not observed [31]. In contrast, Soave and collaborators tested CD44 combined with CD24 in adenocarcinomas of the salivary glands, and found a strong correlation with tumour size and lymph node metastasis [21]. Wang et al. observed a strong correlation of high CD44-expression with distant metastasis, histologic pattern, perineural-invasion, vascular-invasion, and clinical stage [32]. Regarding HNSCC, diverging results were described. An older study by Mack and Gires specifically investigated the expression pattern of CD44 variants (CD44s and CD44v6) in HNSCC, and found no difference between benign and malignant tissue [33]. Whereas several recent studies observed a high expression of CD44 [34–36]. Concerning other tumour entities, the CD44 expression and its prognostic significance are well-studied, e.g. in breast cancer, colorectal cancer, and endometroid cancer [37, 38].

Investigating Nanog, our cohort showed a predominantly low expression with no significant correlation upon survival-rates or clinicopathological parameters. Solely a trend to a positive perineural-invasion was observed. Two other studies showed similar results investigating patients with adenoidcystic or mucoepidermoid-carcinoma [23, 31]. In contrast, Destro Rodrigues and collaborators observed a high expression of Nanog correlated with a perineural-invasion in patients with mucoepidermoid-carcinoma [27]; an association with survival-estimates was not observed. Investigations of other tumour entities showed promising prognostic values of Nanog in endometrioid carcinoma [37], liver cancer, lung cancer, and HNSCC [18].

In our cohort, SOX2 showed a low expression with no correlation to survival-estimates. Regarding clinicopathological parameters, an association with a negative perineural status was observed. Other studies found a high expression of SOX2 together with a correlation with clinical staging. Xu et al. did not observe any correlations when analysing SOX2 alone. However, a high expression of SOX2 in combination with CD44/CD133 showed a significant lower OS [31]. Dai and co-workers found a significant correlation of high SOX2 expression with advanced T-category, distant metastasis, OS, and DFS in patients with adenoidcystic carcinoma [39]. Sedassari et al. found an association with T-category, distant metastasis, grading, recurrence rate, and adverse outcome in their cohort of 30 patients with carcinoma ex-pleomorphic adenoma [40]. In terms of other tumour entities, a prognostic correlation of SOX2 in colon cancer [41], breast cancer [42], and HNSCC [3] is well-documented.

The expression of stem cell markers such as ALDH1, BMI-1, CD44, Nanog, and SOX2 is well-investigated for other tumour entities, and has shown promising results as prognostic parameters with potential as a target for cancer treatment [43, 44]. Malignoma of the salivary glands is rare per se, with rather worse prognosis [1]. Retrospective analyses in literature have found clinical staging as a main prognostic factor [4, 21, 45]. Clinical prospective studies do not exist due to the low incidence of the disease. Regarding clinicopathological studies, 9 studies investigated a correlation of certain CSCs with survival-rates and clinical stages to date (Table 4). Unfortunately, the heterogeneity of cohorts, applied methods, and investigated CSCs differ significantly, therefore, a sound conclusion of those diverging results seems impossible. Regarding the results of our study and the literature, the CSCs ALDH1, BMI-1, CD44, Nanog, and SOX2 seem not to serve as reliable prognostic parameters in adenocarcinomas of the salivary glands.

## Conclusion

In conclusion, our study found no significant correlation of the investigated CSCs ALDH1, BMI-1, CD44, Nanog, and SOX2 with survival-estimates in adenocarcinoma of the major salivary glands. However, observed were a high ALDH1-expression associated with higher grading, a high CD44-expression with localization of the neoplasm, advanced pT- and pN-category, and UICC-stage, as well as a high SOX2-expression with negative perineural invasion.

Recapitulating the results of our study in conjunction with the literature, the CSCs ALDH1, BMI-1, CD44, Nanog, and SOX2 do not seem to serve as reliable novel prognostic parameters in the treatment of adenocarcinoma of the salivary glands.

## Electronic supplementary material

Below is the link to the electronic supplementary material.

Supplementary file1 (PDF 1005 kb)

Supplementary file2 (DOCX 21 kb)

## Data Availability

Original data are available on demand.

## Code availability

Statistical analysis was performed with the software Statistica, version 13.1 (StatSoft Europe, Hamburg, Germany). The respective codes are available on demand.
