# Supplementary figures and images for: Cancer stem cell markers in adenocarcinoma of the salivary glands - reliable prognostic markers?
Source: Eur Arch Otorhinolaryngol. 2020 Oct 3;278(7):2517–28. doi: 10.1007/s00405-020-06389-7 (PMC8165058; doi:10.1007/s00405-020-06389-7)

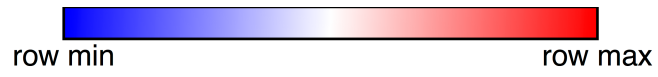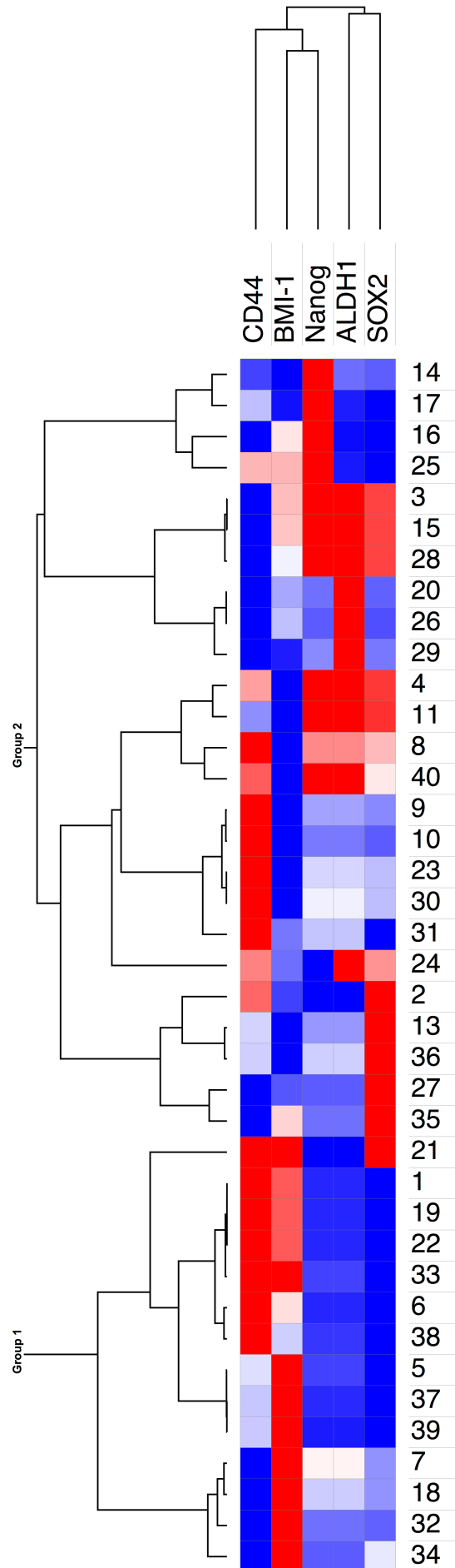

Supplement: Supplementary file 1 — Supplementary file1 (PDF 1005 kb) [file 405_2020_6389_MOESM1_ESM.pdf]
