# Supplementary material for: Cancer stem cell markers in adenocarcinoma of the salivary glands - reliable prognostic markers?
Source: Eur Arch Otorhinolaryngol. 2020 Oct 3;278(7):2517–28. doi: 10.1007/s00405-020-06389-7 (PMC8165058; doi:10.1007/s00405-020-06389-7)
Supplement: Supplementary file 2 — Supplementary file2 (DOCX 21 kb) [file 405_2020_6389_MOESM2_ESM.docx]

| *Supplemental Table 1: Correlation of Group 1 and 2 with Patient and Disease Characteristics* | | | |
| --- | --- | --- | --- |
|  | | | |
|  | Group 2 | Group 1 | *p*-value |
| Sex |  |  |  |
| female | 7 | 3 | 0.652 |
| male | 18 | 11 |  |
| Age |  |  |  |
| < 60 | 10 | 7 | 0.546 |
| ≥ 60 | 15 | 7 |  |
| Localization |  |  |  |
| parotid gland | 19 | 8 | 0.221 |
| submandibular gland | 6 | 6 |  |
| Grading |  |  |  |
| G1/2 | 18 | 10 | 0.970 |
| G3 | 7 | 4 |  |
| Grading |  |  |  |
| G1 | 7 | 0 | **0.029** |
| G2/3 | 18 | 14 |  |
| T-category |  |  |  |
| T1–T2 | 13 | 7 | 0.905 |
| T3–T4 | 12 | 7 |  |
| N-category |  |  |  |
| negative | 14 | 6 | 0.431 |
| positive | 11 | 8 |  |
| Perineural invasion |  |  |  |
| Pn0 | 18 | 8 | 0.345 |
| Pn1 | 7 | 6 |  |
| UICC stage |  |  |  |
| I–II | 10 | 4 | 0.475 |
| III–IV | 15 | 10 |  |

G, grading; N, nodal status; Pn, perineural invasion; T, tumor size; UICC, International Union Against Cancer (7th edition);

Supplemental Table 2: Multivariate Analysis of CSCs with Patients and Disease Characteristics

| CSC | Model fitted | Variable | Estimate | *p*-value |
| --- | --- | --- | --- | --- |
| **ALDH1** | Grading + age | Intercept | 3.008 | 0.275 |
|  |  | Grading-2 | -21.167 | 0.997 |
|  |  | Grading-3 | -20.739 | 0.998 |
|  |  | Age | -0.056 | 0.245 |
|  |  |  |  |  |
| **BMI-1** | pN + grading | Intercept | -0.649 | 0.386 |
|  |  | pN-positive | 1.045 | 0.163 |
|  |  | Grading-2 | 0.460 | 0.609 |
|  |  | Grading-3 | -0.706 | 0.521 |
|  | pT + pN | Intercept | -0.328 | 0.631 |
|  |  | pT-2 | 0.000 | 1.000 |
|  |  | pT-3 | -0.235 | 0.813 |
|  |  | pT-4 | -0.883 | 0.363 |
|  |  | pN-positive | 1.128 | 0.128 |
|  |  |  |  |  |
| **CD44** | Localization + pN + age | Intercept | -1.733 | 0.403 |
|  |  | Localization-2 | -19.283 | 0.995 |
|  |  | pN-positive | 1.900 | 0.995 |
|  |  | Age | -0.046 | 0.146 |
|  | UICC stage + pN | Intercept | -19.570 | 0.996 |
|  |  | UICC stage-2 | 0.000 | 1.000 |
|  |  | UICC stage-3 | -19.220 | 0.996 |
|  |  | UICC stage-4 | 19.350 | 0.996 |
|  |  | pN-positive | 0.129 | 0.895 |
|  | pT + pN | Intercept | -2.668 | **0.023** |
|  |  | pT-2 | -16.346 | 0.994 |
|  |  | pT-3 | 2.086 | 0.109 |
|  |  | pT-4 | 2.140 | 0.086 |
|  |  | pN-positive | 1.166 | 0.202 |
|  |  |  |  |  |
| **Nanog** | Perineural Invasion + localization | Intercept | -2.773 | **0.007** |
|  |  | Perineural invasion-1 | 1.179 | 0.146 |
|  |  | Localization-2 | -17.500 | 0.995 |
|  |  |  |  |  |
| **SOX2** | Perineural invasion + localization + age | Intercept | 1.916 | 0.296 |
|  |  | Perineural anvasion -1 | -18.240 | 0.995 |
|  |  | Localization -2 | -0.655 | 0.529 |
|  |  | Age | -0.046 | 0.120 |
|  | Grading + age | Intercept | 1.916 | 0.296 |
|  |  | Grading-2 | 0.573 | 0.607 |
|  |  | Grading-3 | -17.49 | 0.996 |
|  |  | Age | -0.047 | 0.109 |
|  | UICC stage + age | Intercept | -14.968 | 0.995 |
|  |  | UICC stage-2 | 17.541 | 0.993 |
|  |  | UICC stage-3 | 18.494 | 0.993 |
|  |  | UICC stage-4 | 18.581 | 0.993 |
|  |  | Age | -0.075 | 0.021 |

CSC, cancer stem cell marker; G, grading; N, nodal status; Pn, perineural invasion; T, tumor size; UICC, International Union Against Cancer (7th edition);

Supplemental Table 3: Correlation of Group 1 and 2 with Survival Rates

|  |  | OS (%) | | | DSS (%) | | | RFS (%) | | | LCR (%) | | |
| --- | --- | --- | --- | --- | --- | --- | --- | --- | --- | --- | --- | --- | --- |
|  | n | 3 years | 5 years | *p*-value | 3 years | 5 years | *p*-value | 3 years | 5 years | *p*-value | 3 years | 5 years | *p*-value |
| Group 2 | 25 | 64.9 | 64.9 | 0.329 | 80.8 | 80.8 | 0.980 | 70.5 | 70.5 | 0.545 | 85.2 | 85.2 | 0.880 |
| Group 1 | 14 | 80.8 | 69.3 |  | 82.9 | 82.9 |  | 72.7 | 72.7 |  | 83.1 | 83.1 |  |

DSS, disease specific survival; LCR, local control rate; OS, overall survival; RFS, recurrence free survival;
